# Supplementary material for: The level of postoperative care influences mortality prediction by the POSPOM score: A retrospective cohort analysis
Source: PLoS One. 2021 Sep 29;16(9):e0257829. doi: 10.1371/journal.pone.0257829 (PMC8480745; doi:10.1371/journal.pone.0257829)
Supplement: S1 Table — (DOCX) [file pone.0257829.s001.docx]

**Table S1: Variables included in the POSPOM score**

| Parameter | POSPOM Points |
| --- | --- |
| Age 18-20 | +0 |
| Age 21-25 | +1 |
| Age 26-30 | +2 |
| Age 31-35 | +3 |
| Age 36-40 | +4 |
| Age 41-45 | +5 |
| Age 46-50 | +6 |
| Age 51-55 | +7 |
| Age 56-60 | +8 |
| Age 61-65 | +9 |
| Age 66-70 | +10 |
| Age 71-75 | +11 |
| Age 76-80 | +12 |
| Age 81-85 | +13 |
| Age 86-90 | +14 |
| Age 91-95 | +15 |
| Age >95 | +16 |
|  |  |
| Comorbidities: |  |
| Ischemic heart disease | +1 |
| Cardiac arrhythmia or heart blocks | +1 |
| Chronic heart failure or cardiomyopathy | +4 |
| Peripheral vascular disease | +1 |
| Dementia | +2 |
| Cerebrovascular disease | +1 |
| Hemiplegia | +4 |
| Chronic obstructive pulmonary disease | +1 |
| Chronic respiratory failure | +3 |
| Chronic alcohol abuse | +4 |
| Cancer | +4 |
| Diabetes | +1 |
| Transplanted organ(s) | +2 |
| Preoperative chronic hemodialysis | +1 |
| Chronic renal failure | +2 |
|  |  |
| Index surgery: |  |
| Endoscopic digestive | +0 |
| Ophthalmologic | +0 |
| Gynecologic | +6 |
| Other orthopedic | +6 |
| Interventional cardiorhythmology | +8 |
| Arthroplasty and spine | +9 |
| Ear, nose and throat (ENT) | +9 |
| Minor urologic | +9 |
| Plastic | +9 |
| Major urologic | +12 |
| Others surgery | +12 |
| Minor hepatic | +12 |
| Minor gastrointestinal | +13 |
| Renal transplant | +13 |
| Minor vascular | +13 |
| Orthopedic trauma | +14 |
| Major hepatic | +15 |
| Thoracic | +15 |
| Neuro | +15 |
| Major vascular | +16 |
| Major gastrointestinal | +16 |
| Interventional neuroradiology | +17 |
| Cardiac | +17 |
| Transplant | +22 |
| Multiple trauma related | +22 |
