# Supplementary material for: The level of postoperative care influences mortality prediction by the POSPOM score: A retrospective cohort analysis
Source: PLoS One. 2021 Sep 29;16(9):e0257829. doi: 10.1371/journal.pone.0257829 (PMC8480745; doi:10.1371/journal.pone.0257829)
Supplement: S2 Table — (DOCX) [file pone.0257829.s002.docx]

**Table S2: Characteristics of patient age and comorbidities**

| Demographics |  |  |  |  |
| --- | --- | --- | --- | --- |
| Age (yr; mean, SD, median) | 56.4 | 18.5 | 59 |  |
| Male sex (%, n) | 50.8 | 101,117 |  |  |
|  |  |  |  |  |
| Medical history | **n** | **In-hospital death** | **Mortality (%)** | **Proportion (%)** |
| Ischemic Heart Disease | 22,973 | 1,294 | 5.63 | 11.50 |
| Cardiac arrythmia or heart block | 10,253 | 784 | 7.65 | 5.13 |
| Peripheral vascular disease or abdominal aortic aneurysm | 9,712 | 681 | 7.01 | 4.86 |
| Cerebrovascular disease | 2,429 | 113 | 4.65 | 1.22 |
| Chronic obstructive pulmonary disease | 7,536 | 471 | 6.25 | 3.77 |
| Diabetes | 19,567 | 843 | 4.31 | 9.79 |
| Preoperative chronic hemodialysis | 1,347 | 117 | 8.69 | 0.67 |
| Dementia | 1,971 | 141 | 7.15 | 0.99 |
| Transplanted organs | 1,972 | 88 | 4.46 | 0.99 |
| Chronic renal failure | 10,562 | 705 | 6.67 | 5.29 |
| Chronic respiratory failure | 644 | 66 | 10.25 | 0.32 |
| Chronic heart failure or cardiomyopathy | 8,574 | 399 | 4.65 | 4.29 |
| Hemiplegia | 7,774 | 494 | 6.35 | 3.89 |
| Chronic alcohol abuse | 4,523 | 283 | 6.26 | 2.26 |
| Cancer | 41,009 | 1,181 | 2.88 | 20.53 |
