# Supplementary material for: The level of postoperative care influences mortality prediction by the POSPOM score: A retrospective cohort analysis
Source: PLoS One. 2021 Sep 29;16(9):e0257829. doi: 10.1371/journal.pone.0257829 (PMC8480745; doi:10.1371/journal.pone.0257829)
Supplement: S3 Table — (DOCX) [file pone.0257829.s003.docx]

**Table S3: Distribution of POSPOM score values across surgical disciplines**

| Surgical procedure |  |  |
| --- | --- | --- |
|  | **n** | **Median Score** |
| Cardiac surgery | 10,781 | 32 |
| Interventional neuroradiology | 3,034 | 27 |
| Interventional cardiac rhythmology | 3,562 | 22 |
| Urologic surgery | 9,103 | 21 |
| Vascular surgery | 6,763 | 26 |
| Plastic surgery | 13,760 | 20 |
| Ophtalmology | 22,093 | 10 |
| Gynecologic surgery | 20,694 | 9 |
| Neurosurgery | 21,696 | 25 |
| Ear-nose-throat (ENT) surgery | 23,124 | 16 |
| Orthopedic surgery | 24,833 | 16 |
| Transplant surgery | 392 | 32 |
| Digestive surgery | 13,094 | 24 |
| Liver, biliary tract and pancreas surgery | 3,890 | 25 |
| Thoracic surgery | 2,026 | 27 |
| Endoscopy | 15,043 | 11 |
| Others | 5,370 | 24 |
| Total | 199,258 | 18 |
